# Efficient Photosensitizer Delivery by Neutrophils for Targeted Photodynamic Therapy of Glioblastoma

**DOI:** 10.3390/ph18020276

**Published:** 2025-02-19

**Authors:** Ruojian Wen, Yuwei Liu, Xiang Tian, Yonghong Xu, Xiao Chen

**Affiliations:** 1 Department of Physiology, School of Medicine, Jianghan University, Wuhan 430056, China; 2 Department of Anatomy, School of Medicine, Jianghan University, Wuhan 430056, China; 3 Institute of Biomedical Sciences, School of Medicine, Jianghan University, Wuhan 430056, China; 4 Institute of Ophthalmological Research, Department of Ophthalmology, Renmin Hospital of Wuhan University, Wuhan 430060, China; 5 Department of Pharmacology, School of Basic Medical Sciences, Wuhan University, Wuhan 430072, China

**Keywords:** glioblastoma, photodynamic therapy, neutrophils, targeted delivery

## Abstract

**Background/Objectives:** Glioblastoma (GBM) is the deadliest type of brain tumor and photodynamic therapy (PDT) is a promising treatment modality of GBM. However, insufficient photosensitizer distribution in the GBM critically limits the success of PDT. To address this obstacle, we propose tumoritropic neutrophils (NE) as active carriers for photosensitizer delivery to achieve GBM-targeted PDT. **Methods:** Isolated mouse NE were loaded with functionalized hexagonal boron nitride nanoparticles carrying the photosensitizer chlorin e6 (BNPD-Ce6). In vitro experiments were conducted to determine drug release from the loaded NE (BNPD-Ce6@NE) to mouse GBM cells and consequential photo-cytotoxicity. In vivo experiments were performed on mice bearing intracranial graft GBMs to demonstrate GBM-targeted drug delivery and the efficacy of anti-GBM PDT mediated by BNPD-Ce6@NE. **Results:** BNPD-Ce6@NE displayed good viability and migration ability, and rapidly released BNPD-Ce6 to co-cultured mouse GBM cells, which then exhibited marked reactive oxygen species (ROS) generation and cytotoxicity following 808 nm laser irradiation (LI). In the in vivo study, a single intravenous bolus injection of BNPD-Ce6@NE resulted in pronounced Ce6 distribution in intracranial graft GBMs 4 h post injection, which peaked around 8 h post injection. A PDT regimen consisting of multiple intravenous BNPD-Ce6@NE injections each followed by one extracranial tumor-directed LI 8 h post injection significantly slowed the growth of intracranial graft GBMs and markedly improved the survival of host animals. Histological analysis revealed massive tumor cell damage and NE infiltration in the PDT-treated GBMs. **Conclusions:** NE are efficient carriers for GBM-targeted photosensitizer delivery to achieve efficacious anti-GBM PDT.

## 1. Introduction

Glioblastoma (GBM) is one of the most challenging and treatment-resistant malignancies, with a median overall survival of only 14–20 months despite standard treatment [1,2,3]. The current standard of care of GBM is a maximal surgical resection combined with radiotherapy and adjuvant chemotherapy [1,3]. While the current standard of care is very aggressive, the overall prognosis for GBM patients remains dismal [1,2,3]. Intensive efforts have been focused on developing novel therapies to overcome the unique challenges posed by GBM, including the glioma stem cells, the invasive nature of the tumor, and various physiological and pathological barriers that hampers tumor access by therapeutic agents [3,4,5]. Emerging therapies include immunotherapies such as immune checkpoint inhibitors, chimeric antigen receptor T cells (CAR-T cells), tumor vaccines, targeted molecular agents, and techniques to improve drug delivery across the blood–brain barrier [4,5]. 

Photodynamic therapy (PDT) is a promising GBM treatment that uses a light-sensitive agent, called a photosensitizer, and light to destroy cancer cells [6,7]. Photosensitizer molecules accumulated in tumor cells are excited by light of a specific wavelength and the energized photosensitizer reacts with molecular oxygen to produce singlet oxygen and downstream reactive oxygen species (ROS) that damage the tumor cells, tumor blood vessels, and trigger anti-tumor immune responses [6,7]. Thanks to the short diffusion range (~0.02–1.00 μm) and lifespan (~0.04–4.0 μs) of the singlet oxygen, PDT selectively destroys cancer cells with minimum damage to healthy tissue, which is the key advantage of PDT [6,7,8]. Photosensitizers are a central element of PDT and there have been two generations of photosensitizers in clinical use. However, the success of PDT as a GBM therapy is very limited largely due to demerits of the currently available photosensitizers, such as a lack of tumor targeting, poor tumor tissue penetration, a short plasma half-life, and, particularly, inadequate delivery in the GBM [8,9]. As GBMs are known to actively recruit immune cells circulating in the bloodstream, exploitation of these circulating immune cells as delivery carriers has emerged as a promising strategy to overcome these challenges. 

Neutrophils (NE) are the largest immune cell population patrolling blood circulation and they are the “first responders” to tissue damage. Recent studies have revealed close and complex interactions between neutrophils and GBM [10,11]. NE are actively recruited to the GBM microenvironment through chemotactic signals such as C-X-C motif chemokine ligands 1, 2 and 8 (CXCL1, CXCL2, and CXCL8) secreted by tumor cells, stromal cells, and infiltrating immune cells, allowing selective NE accumulation at the tumor site [11,12]. Once in the tumor, NE can adopt a pro-tumoral phenotype that can damage brain tissue, aid glioma invasion, promote angiogenesis, and inhibit the anti-tumor activity of other immune cells like T cells [11,12,13,14]. In light of these findings, targeting and exploiting NE has become an active area of research for improving GBM therapy. For instance, NE are shown to be able to traverse the blood-brain barrier and infiltrate brain tumors, enabling delivery of therapeutic agents that may otherwise have difficulty reaching the tumor site [15,16]. NE can be loaded with drugs encapsulated in protective liposomes, which can release the payload upon activation of the neutrophils at the tumor site [15,16]. This provides more controlled and localized drug delivery. Studies have shown that NE-mediated delivery of drugs like paclitaxel can significantly improve survival rates in mouse models of GBM compared to treatment with the free drug alone [15,16]. NE can be further engineered with chimeric antigen receptors (CARs) to enhance their tumor-targeting abilities and drug delivery efficiency [17,18]. Overall, NE-based drug delivery holds promise for improving the treatment of GBM and other brain tumors. However, the exploitation of NE in the PDT of GBM is underexplored. 

**Figure 1:**
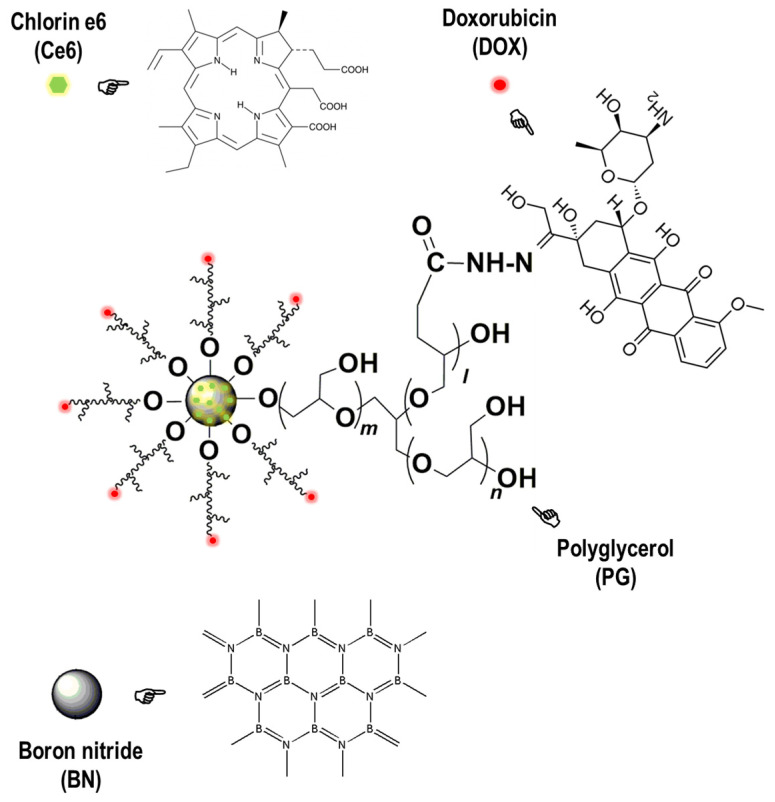
Schematic illustration of the molecular structure of boron nitride–polyglycerol–doxorubicin–Ce6 (BNPD-Ce6).

The current work was carried out aiming to look into the feasibility and effectiveness of using NE to deliver photosensitizers for anti-GBM PDT. For this purpose, a nano-formed photosensitizer based on chlorin e6 (Ce6) carried by functionalized hexagonal boron nitride (BN) nanoparticles (BNPD-Ce6), which we had fabricated and first reported in a previous study [19], were used for delivery by NE. BNPD-Ce6 consists of BN nanoparticles coated with polyglycerol (PG) with attached doxorubicin (DOX) (Figure 1). Ce6 molecules were directly loaded onto the BN. The PG coating provides aqueous solubility and the surface-bound DOX promotes cell uptake of BNPD-Ce6 owing to its affinity for the cell membrane lipid. We first prepared and characterized the BNPD-Ce6-loaded mouse NE (BNPD-Ce6@NE). Drug release from BNPD-Ce6@NE to in vitro mouse GBM cells and consequential photocytotoxicity were next determined. In vivo experiments were then performed on mice bearing intracranial graft GBMs to demonstrate GBM-targeted drug delivery and the efficacy of anti-GBM PDT mediated by BNPD-Ce6@NE.

## 2. Results

### 2.1. Preparation and Characterization of BNPD-Ce6@NE

**Figure 2:**
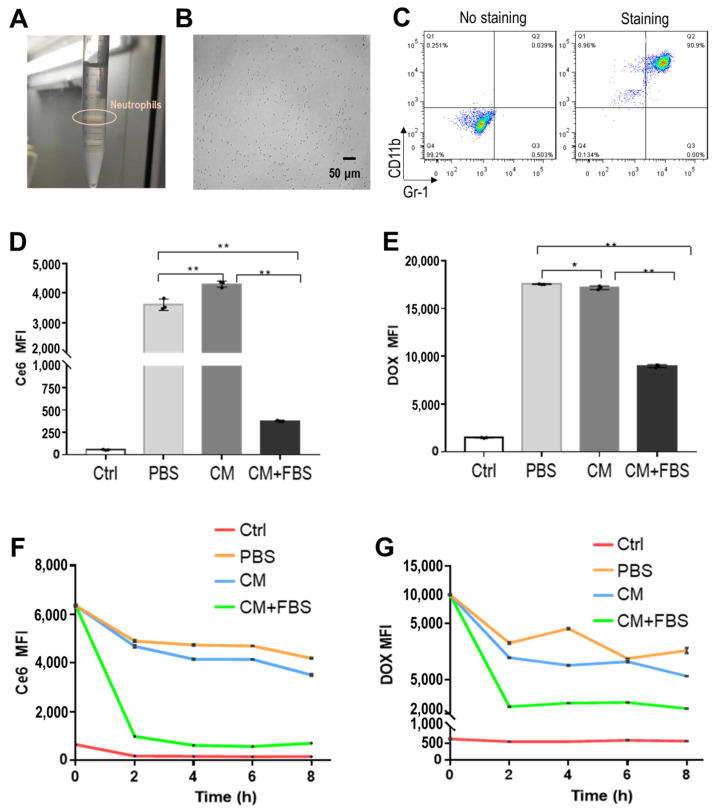
Isolation and characterization of mouse NE and loading of BNPD-Ce6 in the NE. (**A**) Isolated mouse NE in the Percoll gradient solution. (**B**) Isolated mouse NE re-suspended in PBS. (**C**) Immunofluorescent staining of surface CD11b and Gr-1 in isolated mouse NE analyzed by flow cytometry. (**D**,**E**) Loading of BNPD-Ce6 in the NE in different media. (**F**,**G**) Stability of BNPD-Ce6-loaded neutrophils (BNPD-Ce6@NE) in different media. Geometric means were used to quantify fluorescence intensity. (Values were means ± standard deviation (SD), *n* = 3, * *p* < 0.05, ** *p* < 0.01). Representative flow cytometry histograms for (**D**–**G**) are provided in the supplementary material (Figure S2). (Ctrl; Control; MFI: mean fluorescence intensity; FBS: fetal bovine serum).

**Figure 3:**
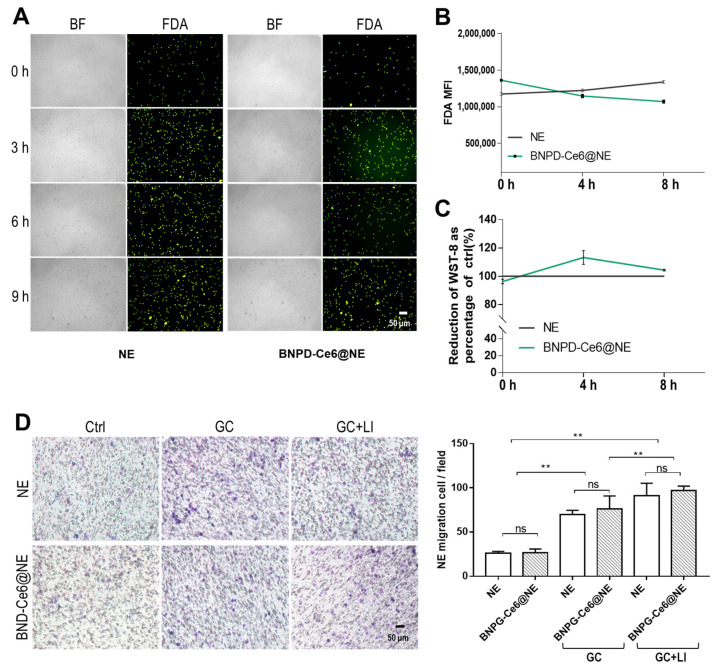
BNPD-Ce6@NE maintained viability and migration ability. (**A**) Fluorescent microscopy of FDA staining in BNPD-Ce6@NE. (**B**) Flow cytometry analysis of FDA staining in BNPD-Ce6@NE. Geometric means were used to quantify fluorescence intensity. (**C**) WST-8 assay of BNPD-Ce6@NE. (**D**) Transwell migration assay of BNPD-Ce6@NE. (Values were means ± standard deviation (SD), *n* = 3, ** *p* < 0.01). Flow cytometry histograms for (**B**) are provided in the supplementary material (Figure S3). (BF: bright field; FDA: fluorescein diacetate staining; NE: neutrophils; BNPD@NE: neutrophils loaded with BNPD; GC: glioblastoma cells; LI: light irradiation; Ctrl: control; ns: no significance).

Mouse NE were isolated using Percoll gradient centrifugation (Figure 2A,B) [20]. The isolated cells were analyzed for the expression of CD11b and Gr-1 (Figure 2C), which are cell surface proteins characteristically expressed in NE. The percentage of CD11b+Gr-1+ cells that were considered to be NE in the isolated cells was >95%. Next, the isolated NE were incubated with BNPD-Ce6 solution in PBS, RPMI 1640 culture medium (CM), and CM with 10% fetal bovine serum (CM+FBS), respectively, for preparation of BNPD-Ce6@NE. As shown in Figure 2D,E, the NE exhibited a high loading capacity of BNPD-Ce6 in PBS and CM but not in CM+FBS. Consistently, BNPD-Ce6@NE were relatively stable in PBS and CM, exhibiting slow spontaneous drug release over an 8-h period, while BNPD-Ce6@NE rapidly discharged the bulk of their payload drug during the first 2 h in CM+FBS. BNPD-Ce6@NE maintained good viability over an 8 h period, as indicated by fluorescein diacetate (FDA) staining and the water-soluble tetrazolium salt-8 (WST-8) cell viability assay (Figure 3A–C). The loading capacity of Ce6 was calculated to be 0.35 µg in 2 × 10^6^ NE in 1 mL of PBS (Figure S1). BNPD-Ce6@NE also exhibited the same migration ability as the non-loaded NE both in the presence and absence of the mouse GL261 GBM cells (GCs), as is indicated by the tanswell migration assay (Figure 3D). Notably, GCs subjected to BNPD-Ce6-mediated photodynamic damage attracted more NE (either loaded with BNPD-Ce6 or not) than the undamaged GCs (Figure 3D).

### 2.2. BNPD-Ce6@NE Efficiently Delivered Ce6 to In Vitro GCs, Which Exhibited Pronounced Photodamage After 808 nm LI

**Figure 4:**
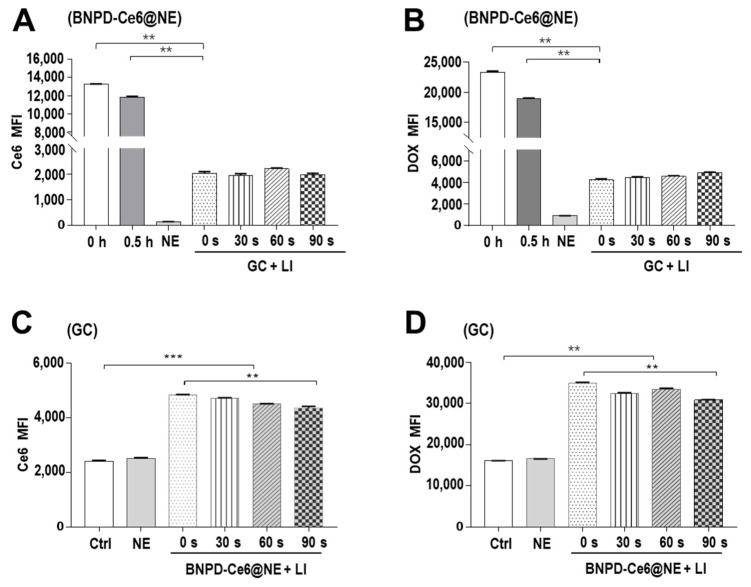
BNPD-Ce6@NE rapidly released BNPD-Ce6 to co-cultured GL261 cells irrespective of LI. BNPD-Ce6@NE were added to in vitro GL261 cells and the mixture was right away subjected to LI (808 nm, 0.5 W/cm^2^) of varied durations (0- 90 s) and 30 min later both BNPD-Ce6@NE and the GL261 cells were taken out for flow cytometry assay of intracellular Ce6 and DOX fluorescence. Geometric means were used to quantify fluorescence intensity. (**A**) Ce6 fluorescence in BNPD-Ce6@NE. (**B**) DOX fluorescence in BNPD-Ce6@NE. (**C**) Ce6 fluorescence in the GL261 cells. (**D**) DOX fluorescence in the GL261 cells. (Values were means ± standard deviation (SD), *n* = 3, ** *p* < 0.01, *** *p* < 0.001). Representative flow cytometry histograms for (**A**–**D**) are provided in the supplementary material (Figure S4). (MFI: mean fluorescence intensity; Ctrl: control).

**Figure 5:**
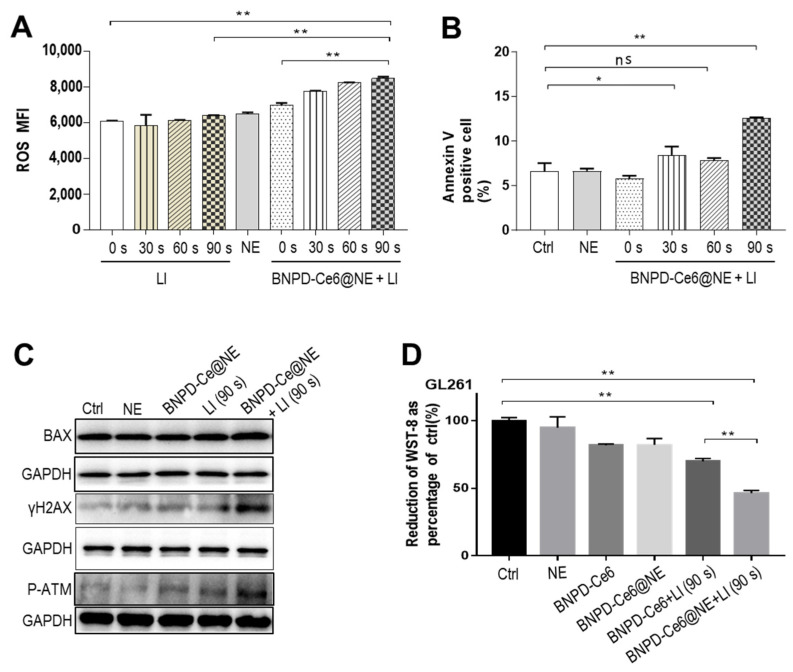
LI elicited toxic responses in GL261 cells following co-culture with BNPD-Ce6@NE. GL261 cells were incubated with BNPD-Ce6@NE for 1 h and then received LI (808 nm, 0.5 W/cm^2^) for varied durations. (**A**) Intracellular ROS content was assayed by DCFH-DA staining and flow cytometry immediately after LI. (**B**) Cell surface expression of Annexin V (marker of apoptosis) was assayed by immunofluorescent staining and flow cytometry 5 h after LI. (**C**) Protein contents of BAX (an indicator of apoptosis), γH2AX, and p-ATM (indicators of DNA damage) were assayed by Western blotting 5 h after LI. (**D**) Cell viability assayed by the WST-8 test. Geometric means were used to quantify fluorescence intensity. (Values were means ± standard deviation (SD), *n* = 3, * *p* < 0.05, ** *p* < 0.01). Representative flow cytometry histograms for (**A**,**B**) are provided in the supplementary material (Figure S5). (DCFH-DA: 2′,7′-dichlorodihydrofluorescein diacetate; MFI: mean fluorescence intensity; Ctrl: control; ns: no significance).

Next, we added BNPD-Ce6@NE to in vitro GCs and the ensuing mixture was immediately subjected to LI (808 nm, 0.5 W/cm^2^) of varied durations (0–90 s). Both BNPD-Ce6@NE and the GCs were taken out for drug content analysis after a 30-min incubation period. As shown in Figure 4, BNPD-Ce6@NE displayed a sharp loss of both of Ce6 and DOX, and in the meantime, the GCs exhibited a consequential gain of Ce6 and DOX, indicating offloading of BNPD-Ce6 from BNPD-Ce6@NE to the GCs. Notably, the transfer of BNPD-Ce6 was neither dependent on nor susceptible to the LI. Then, we applied LI (808 nm, 0.5 W/cm^2^) of varied durations to in vitro GCs that had been incubated with BNPD-Ce6@NE for 1 h. As shown in Figure 5A, LI elicited ROS generation in the BNPD-Ce6@NE-treated GCs in a duration-dependent manner, with the highest ROS production seen with 90 s of LI. Consequently, 90 s of LI engendered pronounced toxicity to the GCs, as indicated by the enhanced surface Annexin V expression, increased bcl-2–associated X (BAX), H2A histone family member X (γH2AX), and phosphorylated ataxia-telangiectasia mutated (P-ATM) expression, and decreased cell viability (Figure 5B–D). 

### 2.3. BNPD-Ce6@NE Efficiently Delivered Ce6 in Intracranial Graft GBMs

**Figure 6:**
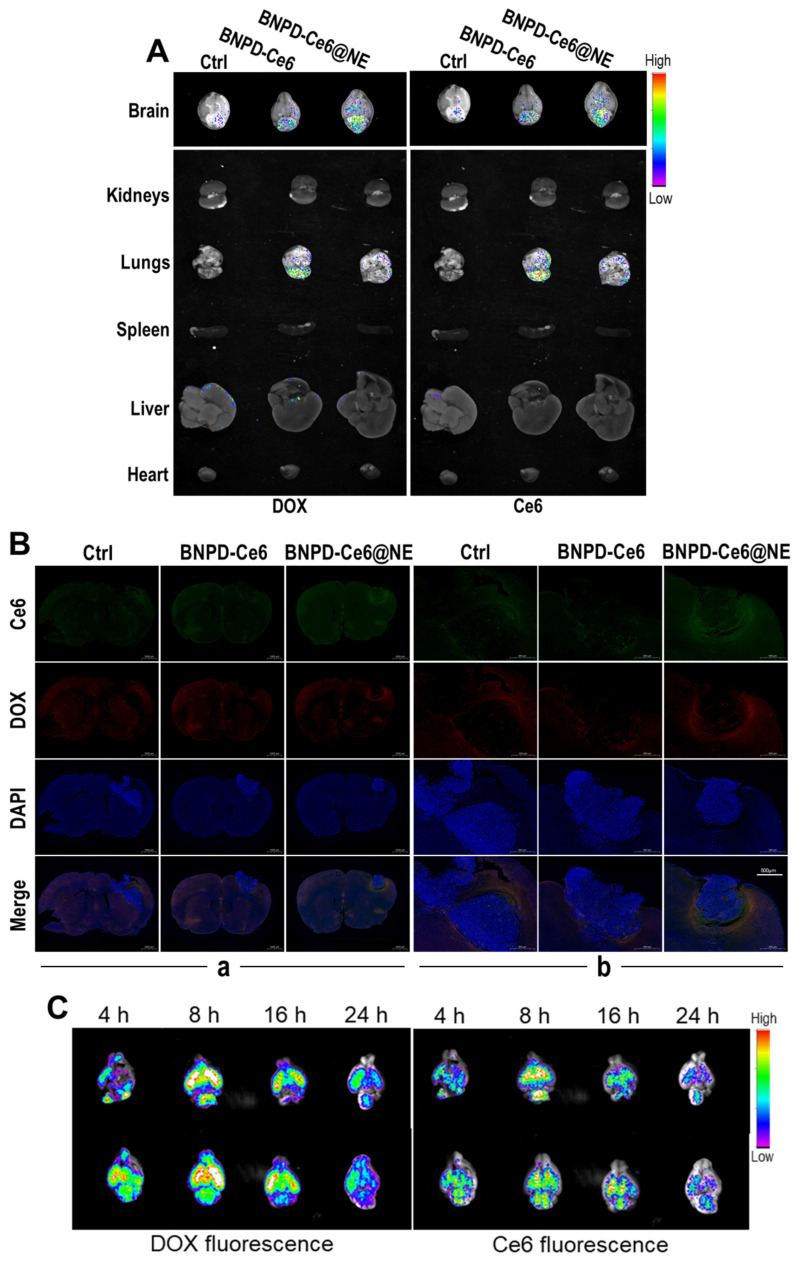
BNPD-Ce6@NE-mediated drug delivery in intracranial graft GBMs. Mice bearing intracranial GL261 graft GBMs were injected with PBS, BNPD-Ce6, or BNPD-Ce6@NE via the tail vein. The animals were sacrificed 4 h post injection and vital organs including the brain, heart, liver, spleen, lungs, and kidneys were taken for ex vivo fluorescent imaging. (**A**) Brain tissue sections were then prepared for fluorescent microscopy. (**B**) Images in part (**a**) show cross sections of whole brains with GBMs. Images in part (**b**) show the GBM locations in the brains. Mice bearing intracranial graft GBMs were injected with BNPD-Ce6@NE via the tail vein. Two animals were sacrificed 4, 8, 16, and 24 h post injection, respectively, and the brains were taken for ex vivo fluorescent imaging (**C**). (Ctrl: control).

To test the in vivo drug delivery capacity of BNPD-Ce6@NE, we intravenously injected BNPD-Ce6@NE in mice bearing intracranial GBMs of grafted GL261 cells. Ex vivo fluorescent imaging of harvested brains (Figure 6A) and fluorescent microscopy of the GBM tissue sections (Figure 6B) revealed appreciable DOX and Ce6 fluorescence in the grafted GBMs 4 h post injection, indicating delivery of BNPD-Ce6 in the GBMs. Notably, an insignificant amount of DOX and Ce6 fluorescence was detected in vital organs including the heart, liver, spleen, lungs, and kidneys, indicating the tumor targeting property of BNPD-Ce6@NE (Figure 6A). We further showed that drug distribution in the GBM peaked at 8 h after the BNPD-Ce6@NE injection (Figure 6C). On the grounds of these observations, we chose to apply LI to the GBMs 8 h after the BNPD-Ce6@NE injection in the subsequent therapeutic study.

### 2.4. BNPD-Ce6@NE Combined with 808 nm LI Achieved Potent Anti-GBM Efficacy

**Figure 7:**
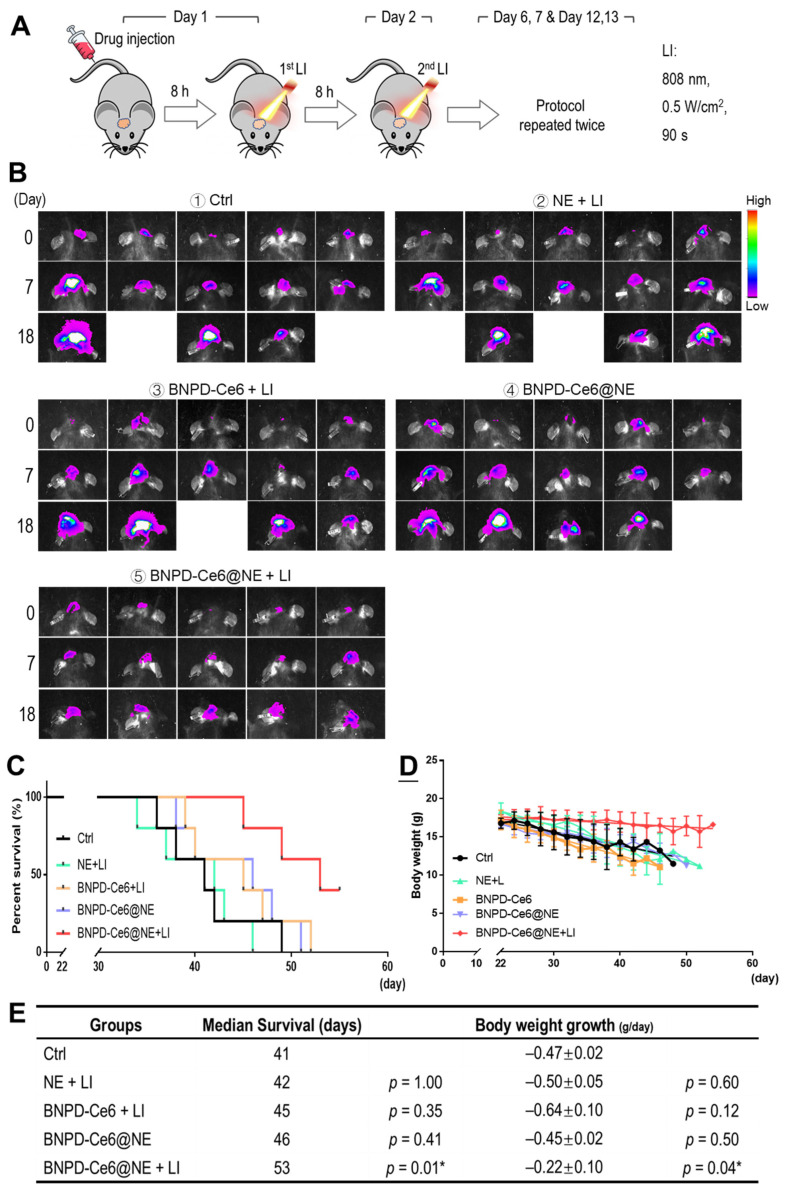
Therapeutic efficacy of GBM PDT mediated by BNPD-Ce6@NE. (**A**) Experimental protocol. Briefly, GBM-bearing mice were divided into 5 groups which were given intravenous injections of PBS (①), NE in PBS (②), BNPD-Ce6 in PBS (③), and BNPD-Ce6@NE in PBS (④, ⑤) according to designation. Starting from 8 h post injection, animals in groups ②, ③, and ⑤ were subjected to 2 extracranial tumor-directed LI (808 nm, 0.5 W/cm^2^, 90 s) at an interval of 8 h. The treatment took 2 days to complete and was repeated twice. (**B**) Fluorescent images of intracranial GBMs taken on days 0, 7, and 18 into therapy. (**C**) Survival curves of the animals. (**D**) Animal body weight monitored over the duration of therapy. (**E**) Statistics of survival analysis and body weight growth rates. Values are means ± standard deviation (SD) (*n* = 5, each other group compared to Ctrl * *p* < 0.05). (Ctrl: control).

**Figure 8:**
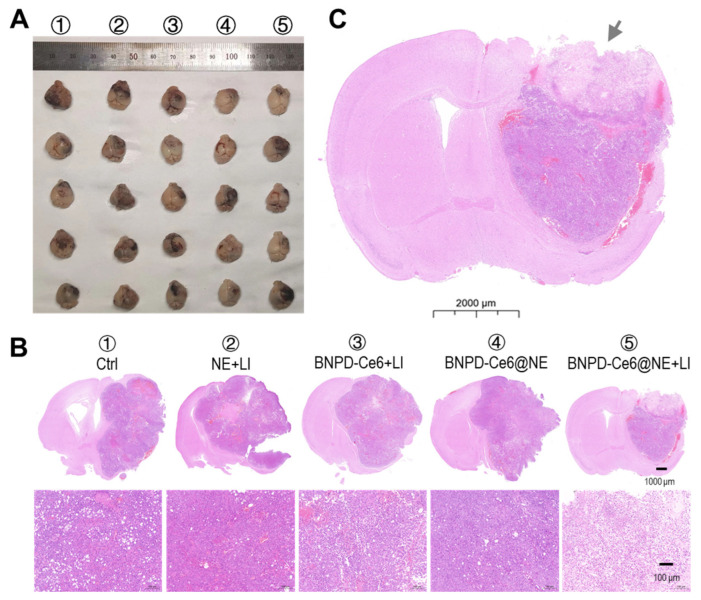
Morphology and histological observation of the intracranial GBMs after therapy. (**A**) Gross examination of resected brains. (**B**) Hematoxylin and eosin staining (H&E) staining of brain tissue sections. (**C**) Higher magnification image of sample ⑤ in (**B**). The area marked by the arrowhead is an area of massive necrosis in a GBM that received BNPD-Ce6@NE + LI.

**Figure 9:**
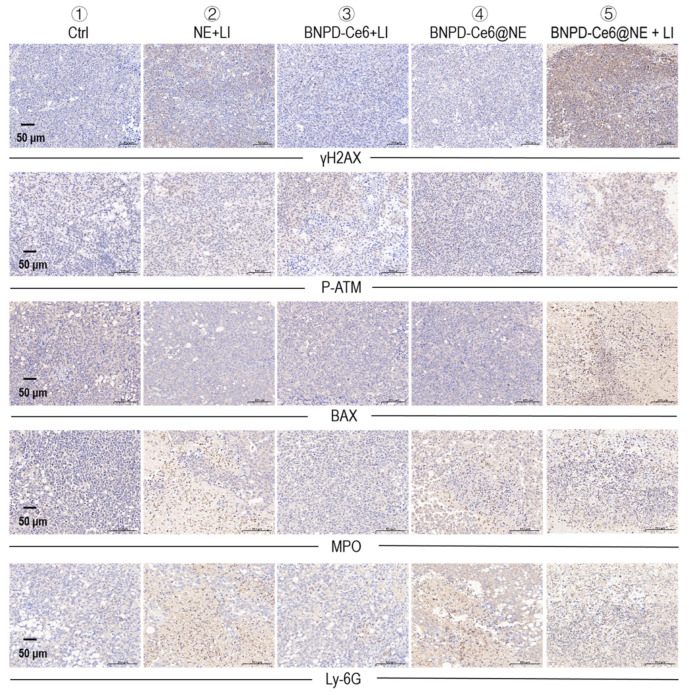
Immunohistochemical staining of H2AX, P-ATM, BAX, MPO, and Ly-6G in the GBMs following treatments.

We subsequently investigated the in vivo anti-GBM efficacy of BNPD-Ce6@NE-mediated PDT in mice bearing intracranial graft GBMs. The experimental protocol is illustrated in Figure 7A. Briefly, the animals were intravenously injected with BNPD-Ce6@NE (with 0.35 μg of Ce6 in 2 × 10^6^ NE in 200 μL of PBS per mouse or 0.0175 mg/kg body weight) and 8 h later each animal received LI (808 nm, 0.5 W/cm^2^, 90 s) at the tumor site. LI was performed a second time 8 h after the first LI. The treatment protocol, i.e., BNPD-Ce6@NE injection plus two applications of LI at the GBM site, was performed on days 1, 2, 6, 7, day 12, and 13. Relevant control groups were set up for comparison. As shown in Figure 7B–E, BNPD-Ce6@NE-mediated PDT (i.e., BNPD-Ce6@NE + LI), as against other treatments, markedly delayed the growth of intracranial graft GBMs and body weight loss and significantly extended survival of the host animals. Notably, two mice that received BNPD-Ce6@NE-mediated PDT were still alive on day 55, while the animals subjected to all other treatments had died by day 52 (Figure 7C). Animal brains either harvested upon death or upon sacrifice at the end of the study are displayed in Figure 8A. H&E staining of brain tissue sections revealed massive necrosis in the GBMs that received BNPD-Ce6@NE-mediated PDT (Figure 8B,C). Note that massive necrosis resulting from BNPD-Ce6@NE-mediated PDT appeared in the outer range of the tumor mass that bore the brunt of the LI likely due to limited issue penetration by the LI, while spontaneous necrosis mostly occurred in the inner part of the tumor mass. See groups ② and groups ⑤ in Figure 8B for comparison. Further IHC staining analysis revealed pronounced expression of γH2AX, P-ATM, and BAX, indicating massive cell damage in the tumors that received BNPD-Ce6@NE-mediated PDT (Figure 9). These tumors also showed signs of heavy neutrophil infiltration, as indicated by the enhanced expression of myeloperoxidase (MPO) and lymphocyte antigen 6 complex locus G6D (Ly-6G) (Figure 9). 

## 3. Discussion

In this work, we have demonstrated that mouse NE can stably carry the nano-formed photosensitizer BNPD-Ce6@NE in quantity. The photosensitizer-loaded NE, i.e., BNPD-Ce6@NE maintained good functional viability and could actively deliver Ce6 in grafted GBM in mice, which eventually led to potent anti-GBM efficacy of PDT. In the process of substantiating the main proposition of exploiting NE to deliver photosensitizer for anti-GBM PDT, we made some findings that are worth discussing. First, we initially had tried to load free Ce6 in the NE only to find a very low loading capacity for free Ce6 (Figure S7). In contrast, the NE displayed a loading capacity a magnitude higher for the nano-formed Ce6, i.e., BNPD-Ce6 than for free Ce6 (Figure S7). This observation was also made in other blood cells such as monocytes, macrophages, and platelets and it is due to this observation that BNPD-Ce6@NE were used throughout this work. Second, FBS was found to have a negative impact on BNPD-Ce6@NE. In the presence of 10% FBS, not only did the NE show a significantly lowered loading capacity of BNPD-Ce6 (Figure S2), but the prepared BNPD-Ce6@NE also exhibited rapid drug release (Figure 2). In view of these observations, BNPD-Ce6@NE were prepared in PBS without FBS for use in the in vitro and in vivo experiments of this work. Nor was FBS used in the in vitro cell experiments. Whether serum can also induce offloading of BNPD-Ce6@NE in mice and humans and to what extent are under investigation. Third, PDT-damaged GCs appeared to attract more BNPD-Ce6@NE (Figure 3D). This observation is in line with NE’s natural tropism towards tissue damage and is a favorable property that will enhance the efficacy of tumor PDT through a positive feedback loop where tumor tissue damage attracts greater BNPD-Ce6@NE infiltration, thus bringing more BNPD-Ce6 into the GBM [21].

The next discussing point is the timing and administering of LI. Ideally, LI should be administered to the GBM when there is maximum photosensitizer accumulation in the GBM. In our work, one intravenous bolus injection of BNPD-Ce6@NE achieved maximum tumor drug accumulation around 8 h post injection and significant tumor drug retention was maintained until 16 h post injection (Figure 6), allowing a relative wide time window for the administering of LI. We had initially considered employing photodynamic action to activate BNPD-Ce6@NE and thereby to trigger active drug offloading. As it later turned out, however, photodynamic action only mildly stimulated offloading of BNPD-Ce6@NE (Figure S6). However, contact with the GCs appeared to provide powerful enough stimuli to trigger rapid offloading of BNPD-Ce6@NE, irrespective of LI (Figure 4). This observation suggests intensive dialogues existing between the GCs and their recruited NE, which can be exploited for NE-mediated tumor drug delivery. Regarding the LI, it is worth noting that we used an 808 nm laser instead of the frequently reported 690 nm laser to elicit photodynamic action mediated by Ce6 and BNPD-Ce6. We have reported this practice in a previously published work [19]. Although Ce6 shows no absorption at 808 nm, this agent can absorb two separated 808 nm photons simultaneously to promote the electronic transition from the ground state to the excited state, thus capacitating the subsequent photodynamic process [22,23]. Recently, chlorin e4 (Ce4), a close analog of Ce6, was shown to be able to generate singlet oxygen and afterglow upon 808 nm excitation [24], which lends support to C6 excitation by an 808 nm laser. These findings favor the use of an 808 nm laser, which has higher tissue penetration than the 690 nm laser, to achieve BNPD-Ce6@NE -mediated PDT. 

Regarding BNPD-Ce6 the photosensitizer, at its core are hexagonal boron nitride (BN) nanoparticles (NPs) composed of nitrogen and boron atoms that are structured similarly to graphene. BN NPs per se lack biological activities and their large specific surface area is agreeable to molecular loading, and surface functionalization can be readily made to BN NPs to accommodate various functionalities [25]. In this study, we used BN NPs (diameter 8–10 nm) with a surface layer of PG decorated with DOX. The PG layer on one hand affords excellent aqueous solubility to the BN NPs and on the other hand suppresses endocytic uptake of the BN NPs by the NE due to the PG’s property to repel contact by cell surface proteins [26]. This issue is overcome through attaching DOX molecules, which have a high affinity for the cell membrane, to the PG using the hydrazone bond. Ce6, the photodynamic effector, was finally loaded in quantity to the modified BN NPs via π-π stacking to yield BNPD-Ce6. BNPD-Ce6 contains DOX, which is a potent chemotherapeutic agent and is supposed to contribute to the anti-GBM efficacy of BNPD-Ce6-mediated PDT. In our work, however, BNPD-Ce6@NE did not exert significant therapeutic efficacy (Figure 7C,E), which might be due to insufficient amounts of DOX delivered to the GBM and low dosing frequency. Although in our work the modified BN NPs served well as the primary photosensitizer carriers to realize NE-mediated, targeted delivery in the GBM, it should be noted that other NPs of different chemical nature and/or with different physical properties can also be used as long as they are compatible with the NE and amenable to drug loading. Finally, it is worth noting that the therapeutic dosage of Ce6 adopted in this study is 0.0175 mg/kg (body weight), far below the frequently used 1–10 mg/kg (body weight) dosage range [27,28], which attests to the capacity and efficiency of NE as carriers for GBM-targeted delivery.

## 4. Materials and Methods

### 4.1. Materials

Boric acid, melamine, bis(4-nitrophenyl) carbonate, and doxorubicin hydrochloride were purchased from Energy Chemical Co., Ltd. (Shanghai, China). Glycidol was obtained from Jiuding Chemical Co., Ltd. (Shanghai, China). Chlorin e6 was purchased from Yuanye Bio-Technology Co., Ltd. (Shanghai, China). Hydrazine monohydrate was obtained from Shanghai Lingfeng Chemicals, Shanghai, China. Anti-γ-H2AX antibody (bs-3185R) was purchased from Bioss, Beijing, China. Anti-p-ATM antibody (GTX132146, GeneTex) and anti-Annexin- V antibody were purchased from Chamot Biotechnology Co., Ltd. (Shanghai, China). Anti-MPO antibody (22225-1-AP) and anti-BAX antibody (50599-2-18) were purchased from Proteintech, Chicago, IL, USA. The ROS Assay Kit was from Beyotime, Shanghai, China, CellROX™. Green reagent was (C10492) from Thermo Fisher Scientific, Waltham, MA, USA, anti-GADPH antibody (PMK053C) from BioPM, Wuhan, China, Fetal bovine serum (FBS) was purchased from QmSuero/Tsingmu Biotechnology, Wuhan, China, RPMI-1640 culture medium (CM) from Servicebio, Wuhan, China, Trypsin EDTA from Beyotime, Shanghai, China, and WST-8 kit from Biosharp, Hefei, China. Fluorescein diacetate (FDA) and 2′,7′-dichlorofluorescein diacetate (DCFDA) were purchased from Sigma-Aldrich, Saint Louis, MO, USA.

### 4.2. BNPD-Ce6 

The synthesis and characterization of BNPD-Ce6 are detailed in a previous work [21] and briefly described in the supplementary information.

### 4.3. Cell Models

GL261-luciferase (GL261-luc) cells and GL261 cells were purchased from the Cell Bank of Shanghai Institutes for Biological Sciences (Shanghai, China). Cells were maintained in RPMI 1640 medium with 10% fetal bovine serum (FBS) (QmSuero/Tsingmu Biotechnology, Wuhan, China). All working cells were maintained in a humidified incubator with 5% CO_2_ at 37 °C. NE from mouse bone marrow were isolated according to a published protocol [20]. This procedure was approved by the Academic Ethics Review Committee of Jianghan University. Briefly, marrow cavities of the tibias and femurs of donor mice were flushed with CM 10% FBS. After hypotonic lysis of the red blood cells, NE were isolated by centrifugation at 500× *g* for 35 min at 28 °C over discontinuous Percoll gradients, consisting of 55 (vol/vol), 65 (vol/vol), and 75% (vol/vol) Percoll in PBS. Cells recovered at the interface of the 65 and 75% fractions were NE which were characterized by the expression of surface Gr-1 and CD11b, markers of NE, via immunofluorescent staining and flow cytometry. NE number was counted using a hemocytometer under a microscope.

### 4.4. Animals 

Female C57BL/6 mice at 4–5 weeks of age (16–20 g) were obtained from the Shanghai Laboratory Animal Center at the Chinese Academy of Sciences (Shanghai, China). Animal handling and experimental procedures were in line with protocols approved by the Animal Care Committee at Jianghan University. 

### 4.5. Preparation and Characterization of BNPD-Ce6@NE

BNPD-Ce6@NE were obtained by incubating isolated NE (2 × 10^6^) with 0.5 μg/mL of BNPD-Ce6 in 1 mL of phosphate-buffered saline (PBS), culture medium (CM), or CM with 10% FBS (CM-FBS) at 37 °C for 1 h. BNPD-Ce6@NE were then pelleted by centrifugation (3 min, 600× *g*), re-suspended in 500 µL of PBS and then immediately examined for drug loading via flow cytometry analysis of cellular fluorescence of Ce6 and DOX. For evaluation of loading stability, BNPD-Ce6@NE prepared in CM as described above were re-suspended in 1.5 mL of PBS, CM, or CM-FBS and incubated at 37 °C. Samples (250 µL of suspension) were taken at 0, 2, 4, 6, and 8 h for flow cytometry analysis of cellular fluorescence of Ce6 and DOX. For an assay of viability, BNPD-Ce6@NE (2 × 10^6^) prepared in CM were re-suspended in 0.5 mL of PBS containing 10 µg/mL of FDA and incubated at 37 °C for 5 min. BNPD-Ce6@NE were then pelleted and re-suspended in 1.5 mL of CM and incubated at 37 °C and samples (200 µL of suspension) were taken at 0, 3, 6, and 9 h for fluorescent microscopy and flow cytometry analysis of cellular fluorescence of fluorescein. Alternatively, BNPD-Ce6@NE (2 × 10^6^) prepared in CM were re-suspended CM and incubated at 37 °C and samples containing 5 × 10^5^ BNPD-Ce6@NE were taken at 0, 4, and 8 h for a WST-8 assay. The migration ability of BNPD-Ce6@NE was evaluated using a Transwell device (insert pore size 8.0 μm, Corning, New York, CA, USA). Briefly, GCs were seeded in the lower chamber at a density of 2.5 × 10^5^ cells/well. After a stabilization period of 24 h, some GCs were treated with 0.5 μg/mL of BNPD-Ce6@NE for 4 h followed by laser irradiation (808 nm, 0.5 W/cm^2^, 90 s). Then, 5 × 10^4^ BNPD-Ce6@NE or NE in 200 µL CM were added into the insert and incubated with the GCs for 6 h. The insert was then taken out, fixed with 4% paraformaldehyde, and stained with 0.1% crystal violet (Beyotime, Shanghai, China,) in PBS. After washing with PBS, the cells attached to the lower side of the bottom membrane were observed using a BDS400-FL fluorescence microscope (Chongqing Optec Instrument Co., Ltd, Chongqing, China) and cells in each of 5–8 fields of view were counted. 

### 4.6. BNPD-Ce6@NE Offloading to In Vitro GL261 Cells 

BNPD-Ce6@NE (4 × 10^5^) were added to GCs (2.5 × 10^5^ cells/well) in 24-well plates. The mixtures were immediately subjected to LI (808 nm, 0.5 W/cm^2^) for 0, 30, 60, or 90 s, and subsequently maintained in CM at 37 °C for 30 min. BNPD-Ce6@NE and the GCs in each well were then separately taken out for flow cytometry analysis of cellular fluorescence of Ce6 and DOX. NE alone, NE in mixture with GCs, and BNPD-Ce6@NE alone were used for control. 

### 4.7. BNPD-Ce6@NE-Mediated Phototocytoxicity of In Vitro GL261 Cells

BNPD-Ce6@NE (4 × 10^5^) were added to GCs (2.5 × 10^5^ cells/well) in 24-well plates. The mixtures were immediately subjected to LI (808 nm, 0.5 W/cm^2^) for 0, 30, 60, and 90 s, and subsequently maintained in CM at 37 °C for 30 min. The supernatant with BNPD-Ce6@NE in each well was then replaced with CM and LI (808 nm, 0.5 W/cm^2^) was applied to the remaining GCs for 0, 30, 60, or 90 s. The treated GCs were harvested 6 h later and Annexin V expression was determined by staining with the Annexin V-FITC reagent (Multisciences, Hangzhou, China) and flow cytometry according to the manufacturer’s protocol. In brief, 2 × 10^5^ cells were re-suspended in 100 µL of binding buffer and 5 µL of Annexin V-FITC reagent was added to each sample, which was then incubated for 15 min at room temperature in the dark. An additional 400 µL of binding buffer was then added to the reaction prior to flow cytometry analysis. The procedure was repeated on GCs pre-stained with DCFDA, which were taken immediately after LI for determination of ROS generation via flow cytometry. The procedure was also repeated in a 96-well format for WST-8 assay of cell viability and in a 6-well format for Western blotting assay of BAX, γH2AX, and P-ATM content. Briefly, the treated GCs were rinsed with ice-cold PBS and then lysed in RIPA buffer with 1% protease inhibitor cocktail (Sigma-Aldrich, Saint Louis, MO, USA). Cell lysates were centrifuged and protein content was determined using a bicinchoninic acid (BCA) assay kit (BL521A, Biosharp, Hefei, China). Equal protein aliquots (25 μg) were fractionated by SDS-PAGE and transferred to a PVDF membrane. The membrane was blocked with 5% fat-free milk in Tris-buffered saline with Tween 20 (TBST) and incubated with antibodies against BAX (50599-2-Ig, Proteintech, Chicago, IL, USA), γH2AX (bs-3185R, Bioss, Beijing, China), P-ATM (GTX132146, GeneTex, Irvine, CA, USA), and GADPH (PMK053, BioPM, Durham, NC, USA) overnight at 4 °C. Protein bands were imaged using a horseradish peroxidase-conjugated secondary antibody and the ECL substrate (Thermo Fisher Scientific, Waltham, MA, USA). The films were exposed with a Bio Imaging system (Syngene, Bangalore, India).

### 4.8. Drug Delivery by BNPD-Ce6@NE to Intracranial Graft GBM in Mice

Mouse intracranial orthotopic GBM models were established to demonstrate GBM-targeted delivery by BNPD-Ce6@NE. To establish intracranial GBM, GL261-luc cells growing in the log phase were re-suspended in PBS (30,000 cells/μL) for intracranial implantation. The establishment of intracranial GBM was performed according to published protocols [19]. Briefly, mice with shaved heads under Nembutal anesthesia (60 mg/kg, 0.1 mL/10 g, intraperitoneal injection) were secured in a stereotactic head holder. A burr hole 1 mm in diameter was made in each mouse’s cranium, 2 mm right lateral of the bregma, 1 mm anterior to the coronal suture, and to a depth of 1.5 mm below the cortical surface. Tumor cells suspended in 6 μL of PBS (30,000 cells/μL) were then slowly injected through the burr hole over 5 min. Tumor growth was monitored by bioluminescent imaging (Bruker In-Vivo Xtreme, Hennigsdorf, Germany). Animals on day 21 post implantation were used in later experiments. For drug delivery experiments, the GBM-bearing mice were randomly assigned to groups (2 mice per group) with designated treatments: ➀PBS (Ctrl), ➁BNPD-Ce6, and ➂BNPD-Ce6@NE. The animals, according to their designation, received intravenous injections of PBS, BNPD-Ce6, or BNPD-Ce6@NE in 200 μL of PBS per mouse. Dosage was 0.35 μg of Ce6 in 2 × 10^6^ NE per mouse or 0.0175 mg/kg body weight for a mouse of 20 g. All animals were sacrificed 4 h after drug injection and brains were harvested, fixed with 4% paraformaldehyde, and cryosections (5 μm) of the brains were prepared for fluorescent microscopy. DAPI counterstaining was performed on the brain sections. Briefly, the tissue sections were covered with 1 µg/mL of DAPI solution in the dark for 5 min at room temperature. Excess stain was removed by gently rinsing the slides twice with PBS (2–3 min per wash). Excess fluid was then blotted and antifade mounting medium was applied to the sections before coverslips were placed. For determination of the timing of LI, 8 GBM-bearing mice were intravenously injected with 2 × 10^6^ BNPD-Ce6@NE in 200 μL of PBS. Two animals were sacrificed at hours 4, 8, 16, and 24, respectively, post injection. The brain of each animal was taken for fluorescent imaging and fluorescent microscopy of tissue sections. 

### 4.9. Anti-GBM PDT Mediated by BNPD-Ce6@NE 

Intracranial GBM grafts of GL261-luc cells were established as described above. On day 20 post-implantation, the GBM-bearing mice were randomly assigned to 5 groups (5 mice per group) with the following treatments: ➀PBS (Ctrl), ➁NE+LI, ➂BNPD-Ce6+LI, ➃BNPD-Ce6@NE, ➄BNPD-Ce6@NE+LI. The animals, according to their designation, received intravenous injections of PBS, NE, BNPD-Ce6, and BNPD-Ce6@NE, respectively, in 200 μL of PBS per mouse. NE number was 2 × 10^6^ wherever NE was involved. Dosage was 0.35 μg of Ce6 per mouse or 0.018 mg/kg body weight for a mouse of 20 g. The animals in groups ➁, ➂, and ➄ received extra-cranial LI (808 nm, 0.5 W/cm^2^, 90 s) at the tumor site at hours 8, 24, and 48 post drug injection. The treatment protocol, which consisted of one drug injection and subsequent two applications of LI, was performed three times (Figure 7A). The animals were inspected daily till they reached a moribund state, at which time they were sacrificed and the brains and vital organs were harvested for immunohistochemical (IHC) staining analysis. Body weight was measured every other day throughout the experiment duration and brain tumor growth was monitored using in vivo fluorescent imaging for the initial 18 days. For in vivo imaging of the brain tumors, mice under Nembutal anesthesia (60 mg/kg, 0.1 mL/10 g, intraperitoneal injection) were given luciferin (D-Luciferin potassium salt, 150 mg/kg, Sciencelight, luc001, Tuling Biotech, Shanghai, China) via intraperitoneal injection, and then imaged 10 min after injection. For IHC staining, paraffin sections (5 μm) of the harvested tissues were dewaxed, rehydrated. Antigen retrieval was performed by incubating with a sodium citrate buffer (10 mM, pH 6) for 20 min, and then incubated with 3% hydrogen peroxide for 10 min at room temperature. The tissue sections were then blocked with 5% bovine serum albumin (BSA) for 30 min, stained with the desired antibodies, washed with PBS, and stained with secondary antibody (PV-9000, ZSGB-BIO, Beijing, China) for 1 h at 37 °C. DAB (ZLI-9018, ZSGB-BIO, Beijing, China) was applied for 5 min at room temperature for coloration. The nuclei were counterstained with hematoxylin. Antibodies for the IHC staining included rabbit anti-γH2AX (Bioss, Beijing, China, bs-3185R, 1:500, overnight incubation at 4 °C), rabbit anti-p-ATM (GeneTex, Zeeland, MI, USA, GTX132146, 1:200, incubation overnight at 4 °C), rabbit anti-BAX (Proteintech, Chicago, IL, USA, 50599-2-Ig, 1:200, overnight incubation at 4 °C), rabbit anti-MPO (Proteintech, Chicago, IL, USA, 22225-1-AP, 1:1000, 1 h incubation at room temperature), and rat anti-Ly-6G (Proteintech, Chicago, IL, USA, 65140-1-Ig, 1:200, 1 h incubation at room temperature). 

### 4.10. Statistical Analysis

The in vitro data are presented as mean ± (standard deviation) SD. One-way analysis of variance (ANOVA) was used to compare between two groups, and two-way ANOVA with post hoc Tukey’s honestly significant difference (HSD) test was used to compare more than two groups. The normality of the data was tested using the D’Agostino–Pearson test. Data were tested for homogeneity of variance using the Bartlett’s and Brown–Forsythe tests. In the in vivo therapy study, the Kaplan–Meier method was used to analyze animal survival and the Gehan–Breslow–Wilcoxon test was used to compare different Kaplan–Meier curves due to the small group size.

## 5. Conclusions

In conclusion, findings from this work provide compelling evidence that NE are efficient drug carriers for photosensitizer delivery in the GBM, which in combination with LI can realize efficacious anti-GBM PDT.

## Data Availability

Data are contained within the article and Supplementary Material.

## Supplementary Materials

The following supporting information can be downloaded at: https://www.mdpi.com/article/10.3390/ph18020276/s1, Figure S1: Quantitation of Ce6 loading capacity in BNPD-Ce6@NE; Figure S2: Representative flow cytometry histograms for Figure 2D–G. Figure S3: Representative flow cytometry histograms for Figure 3B; Figure S4: Representative flow cytometry histograms for Figure 4A–D. Figure S5: Representative flow cytometry histograms for Figure 5A,B; Figure S6: Effect of LI on drug release from BNPD-Ce6@NE; Figure S7: Loading capacity and stability of free Ce6 and BNPD-Ce6 in NE.

## Abbreviations

The following abbreviations are used in this manuscript:

GBM	glioblastoma
PDT	photodynamic therapy
CARs	chimeric antigen receptors
Ce6	chlorin e6
LI	irradiation
ROS	reactive oxygen species
NE	neutrophils
BNPD-Ce6	boron nitride (BN)-based nanoparticles carrying chlorin e6
BNPD-Ce6@NE	BNPD-Ce6-loaded mouse NE
GCs	GBM cells
PDA	photodynamic action
Ce4	chlorin e4
hBN	hexagonal boron nitride
NPs	nanoparticles
