# Supplementary material for: Efficient Photosensitizer Delivery by Neutrophils for Targeted Photodynamic Therapy of Glioblastoma
Source: Pharmaceuticals (Basel). 2025 Feb 19;18(2):276. doi: 10.3390/ph18020276 (PMC11859058; doi:10.3390/ph18020276)
Supplement: Supplementary file 1 [file pharmaceuticals-18-00276-s001.zip › pharmaceuticals-3458731-SI.pdf]

Supplement information

Synthesis and characterization of BNPD-Ce6

Synthesis of BN nanoparticles

BN nanoparticles with a diameter of 8–10 nm were synthesized according reported method [1] with minor modification. Briefly, boric acid (3.71 g, 0.06 mol) and melamine (1.26 g, 0.01 mol) were mixed by grinding, which was then heated to 900 °C with a heating rate of 10 °C/min and maintained for 2 h under nitrogen flow to yield a white solid. Ground BN powder was sonicated in water for 1 h, and the resulting dispersion was centrifuged at 3000 rpm for 10 min. BN nanoparticles in the supernatant were collected by high-speed centrifugation (20,000 rpm, 1 h) for further surface modification.

Synthesis of BN-PG

BN nanoparticles were dispersed in 10 mL of glycidol by bath sonication. The dispersion was heated at 140 °C in an oil bath for 24 h. After being cooled down, the gel was redispersed in 50 mL of water by bath sonication. The crude product was subjected to ultracentrifugation (70,000 rpm, 5 h) to remove free polyglycerol in the supernatant. This process was repeated three times to give BN-PG nanoparticles as a light brown colloid.

Synthesis of BN-PG-PhNO$_2$

BN-PG (0.2 g) was thoroughly dried in vacuum drying oven, and then redispersed in 5 mL of dry DMF by bath sonication. Bis(4-nitrophenyl) carbonate (0.1 g, 3.3 mmol) was dissolved in 5 mL of dry DMF, and then added into the BN-PG dispersion. After that, triethylamine (0.1 mL) was added dropwise. After stirring for 24 h at room temperature, the crude product was collected by centrifugation at 14,000 rpm for 1 h, and then purified by washing/centrifugation with DMF three times.

Synthesis of BN-PG-NHNH$_2$

BN-PG-PhNO$_2$ (0.1 g) was well dispersed in 5 mL of DMF by bath sonication, which was then added with 0.2 mL of hydrazine monohydrate and heated at 90 °C overnight.

The crude product was collected by centrifugation at 14,000 rpm for 1 h, and then purified by washing/centrifugation with DMF three times.

Synthesis of BN-PG-DOX

BN-PG-$NHNH_2$ (30 mg) and doxorubicin hydrochloride (4.0 mg, 0.007 mmol) were mixed in 4 mL of water by bath sonication. The pH of the mixture was adjusted to 7.0 using diluted NaOH. After stirring at 50 °C in the dark for 24 h, the crude product was collected by centrifugal filtration (100 KDa), and then purified by washing with water until the filtrate became colorless.

Synthesis of BN-PG-DOX

BN-PG-DOX (20 mg) was well dispersed in 5 mL of water by bath sonication, which was then mixed with Ce6 (2 mg, 0.0033 mmol) dissolved in 4 mL of 1 × PBS. The mixture was stirred at room temperature in the dark for 24 h. Next, the crude product was collected by centrifugal filtration (100 KDa), and then purified by washing with water until the filtrate became colorless. The product was redispersed in water and stored at 4 °C prior to use.

Reference

1. Xu, H.Z.; Li, T.F.; Ma, Y.; Li, K.; Zhang, Q.; Xu, Y.H.; Zhang, Y.C.; Zhao, L.; Chen, X. Targeted photodynamic therapy of glioblastoma mediated by platelets with photo-controlled release property. Biomaterials. 2022, 290, 121833. DOI: 10.1016/j.biomaterials.2022.121833

A

The standard curve of absorbnace												
Concetraction (g/mL)	73.75	36.875	18.437	9.218	4.609	2.307	1.152	0.576	0.288	0.144	0.0724	0.0569
Absorbance (410nm)	2.295	1.2267	0.6137	0.3529	0.189	0.1136	0.0813	0.0638	0.0578	0.0553	0.0569	0.0495

B

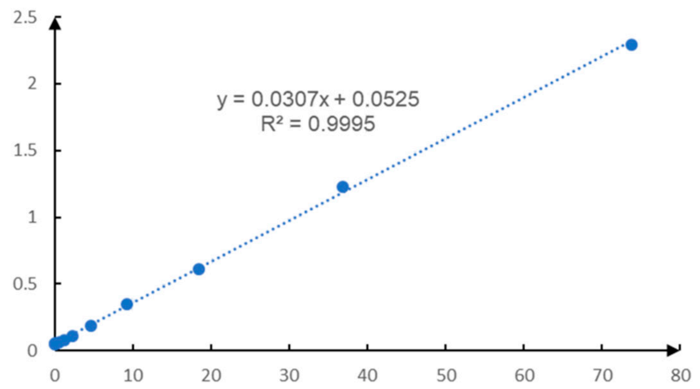

C

Cell	BNPD-Ce6@NE
Measured value	0.0559<br>0.0563<br>0.0595
Mean	0.057233
Calculated concentration (μg / mL)	0.1542
Uptake rate (%)	69.17%

Figure S1. Quantitation of Ce6 loading capacity in BNPD-Ce6@NE. Isolated mouse NE (2×106 /mL) were incubated with 1 mL of PBS containing 0.5 μg/mL of BNPD-Ce6 for 1 hr. The loaded NE were pelleted and the supernatants collected. Ce6 contents in the supernatants were measured by spectrophotometry. (A-B) Standard curve of Ce6 absorbance.(C) Loading capacity of Ce6 was calculated to be 0.35 μg in 2×106 NE in 1 mL of PBS.

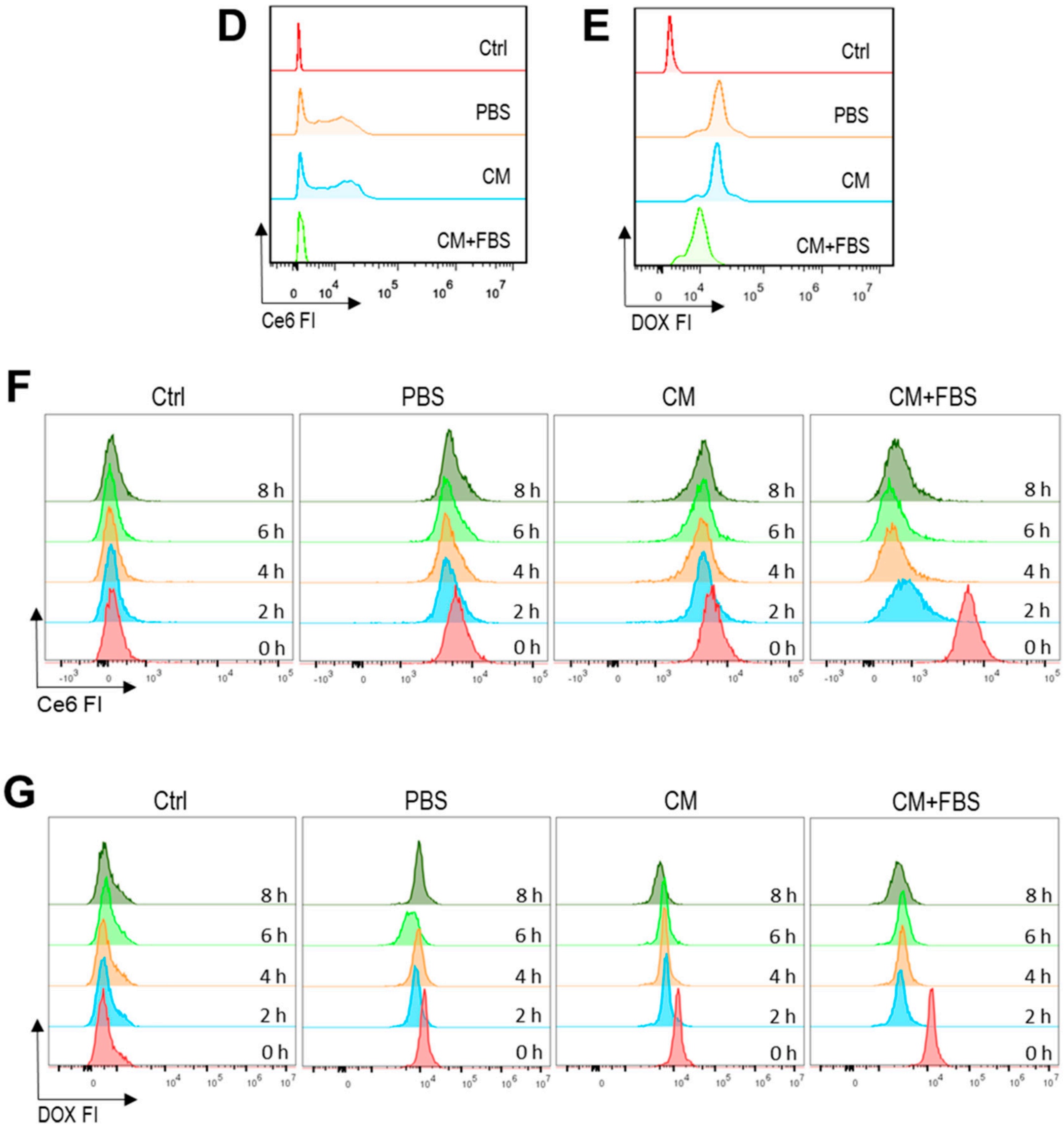

Figure S2. Representative flow cytometry histograms for Figure 2 D-G

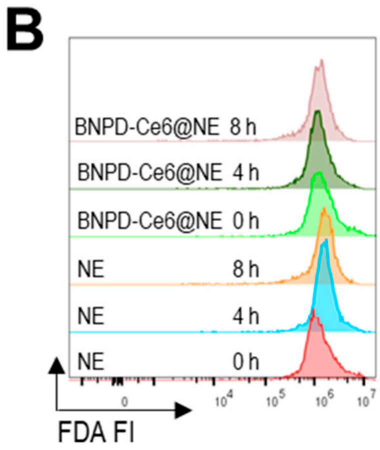

Figure S3. Representative flow cytometry histograms for Figure 3 B.

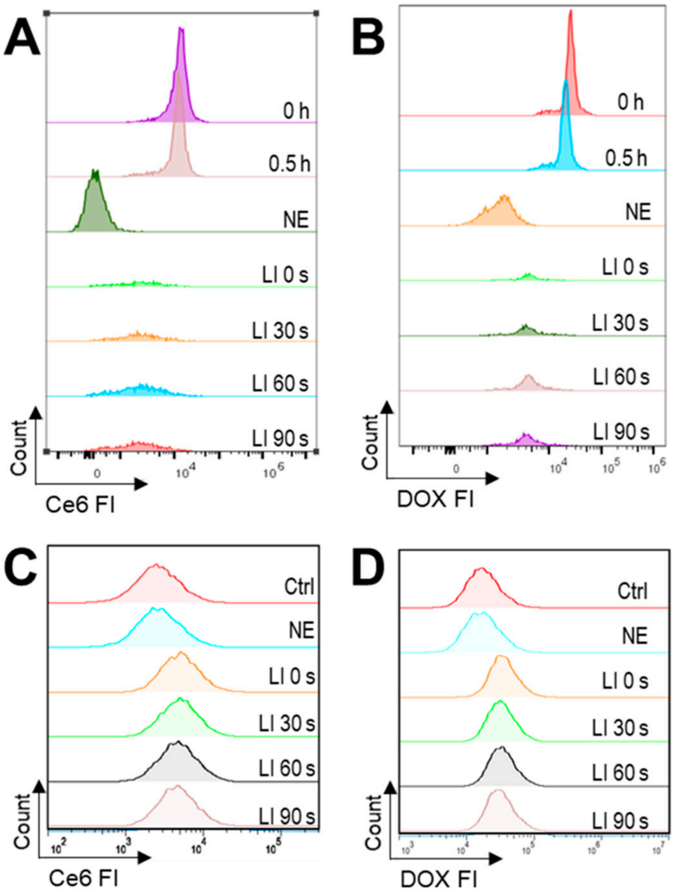

Figure S4. Representative flow cytometry histograms for Figure 4 A-D.

.

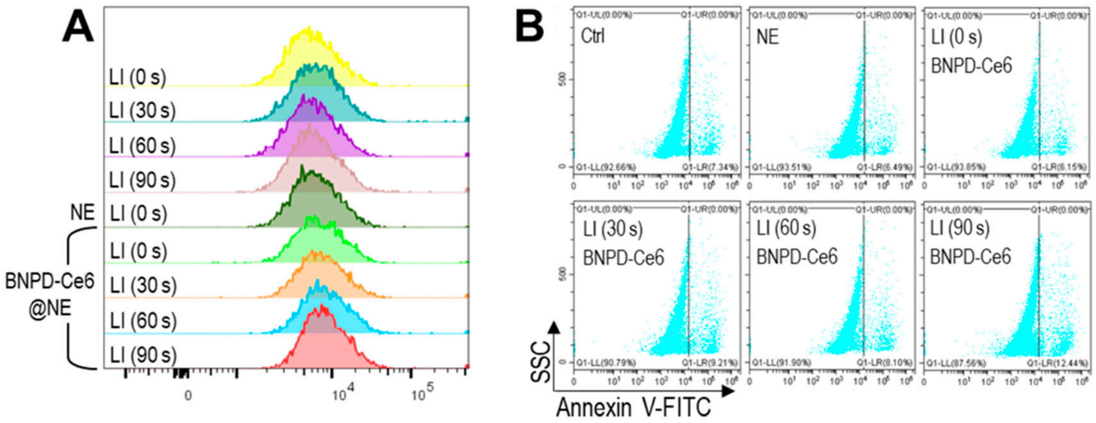

Figure S5. Representative flow cytometry histograms for Figure 5 A-B.

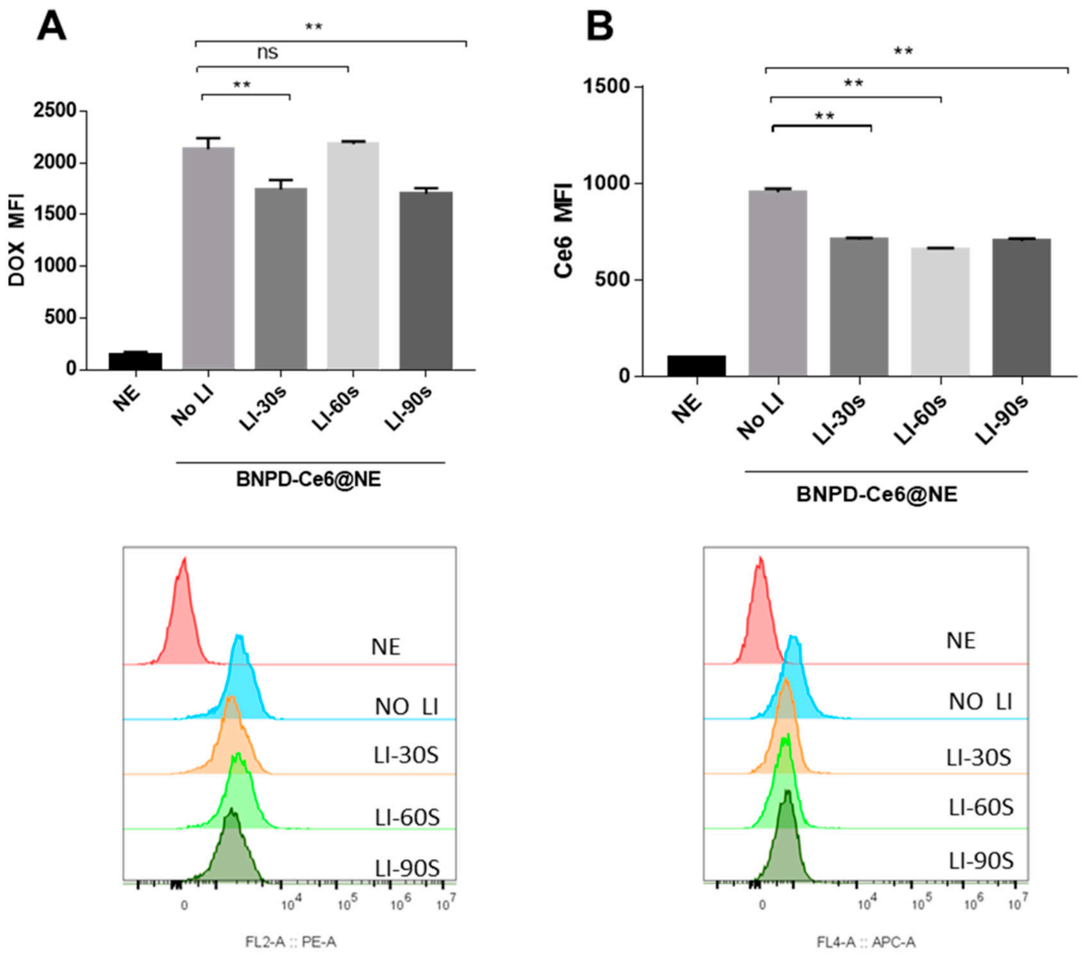

Figure S6. Effect of LI on drug release from BNPD-Ce6@NE. BNPD-Ce6@NE prepared in culture medium (CM) were subjected to LI (808 nm, 0.5 W/cm2) for 30, 60, and 90 s before being taken for evaluation of drug content by flow cytometry. Fluorescent intensity was quantitated using geometric means. Values are mean ± SD (n = 3).

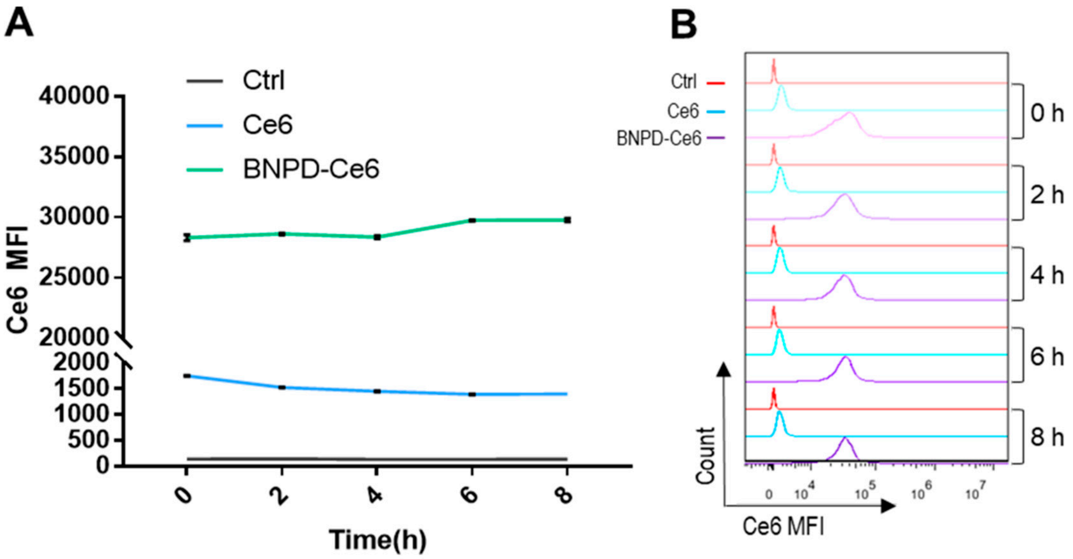

Figure S7. Loading capacity and stability of free Ce6 and BNPD-Ce6 in NE. Isolated mouse NE were incubated with FBS-free culture medium (RPMI 1640) containing 0.5 μg/mL of Ce6 or BNPD-Ce6 for 1 hr and then maintained in FBS-free culture medium at 37 °C for 8 hr. Aliquots of the NE were taken at 2-hr intervals for evaluation of drug content by flow cytometry. Fluorescent intensity was quantitated using geometric means. Values are mean ± SD (n = 3).
